# From Biological Models to Industrial Production: How Industry 4.0 Enables the Manufacturability and Scalability of Biomimetic Design

**DOI:** 10.3390/biomimetics11090614

**Published:** 2026-09-01

**Authors:** Alaeddin Koska

**Affiliations:** Department of Management and Organization, Türkoğlu Vocational School, Kahramanmaraş İstiklal University, Kahramanmaras 46800, Türkiye; alaeddin.koska@istiklal.edu.tr

**Keywords:** biomimetic design, Industry 4.0, manufacturability, scalability, artificial intelligence, additive manufacturing, production management, operational performance

## Abstract

The industrial relevance of biomimetic design rests on whether a biological principle can be converted into an engineering solution that is manufacturable, verifiable, scalable, and traceable in operation. This critical integrative review considers that problem from a production-management perspective and examines the contribution of Industry 4.0 technologies to design, validation, manufacture, and industrial use. The evidence base consists of 50 purposively selected Gold Open Access full texts (*n* = 50) from the Web of Science Core Collection. Each study was coded for its biological model, design principle, digital technology, material and manufacturing route, manufacturability, scale, evidence level, and reported performance. CAD, modeling, simulation, machine learning, optimization, and digital manufacturing support different parts of the transformation process, whereas sensors, robotics, and IIoT supply functional and operational feedback. The direct studies report laboratory- or prototype-level improvements in strength, stiffness, material or support use, drag, thermal and energy performance, sensing, and actuator durability. None, however, demonstrates E4–E5 evidence across the complete chain. Evidence on serial production, process capability, stable quality, total cost, standardization, certification, and life-cycle performance is still limited. Drawing on these findings, the review proposes a technology–production–performance framework and a set of measurable Industry 5.0 indicators. Its main limitations are the Gold Open Access and English-language boundaries, purposive rather than exhaustive selection, unavailable exact search strings and record-level exclusion counts, single-researcher coding, and outcome heterogeneity that prevented meta-analysis.

## 1. Introduction

Biomimetic design is more than the superficial replication of a natural form. Its central engineering challenge is to explain how a biological system performs a specific function through structure, material, process, or feedback, translate that relationship into technical requirements, construct a computable design, and validate the solution within a repeatable manufacturing process. Foundational research describes this as a cross-domain transfer problem: biological solutions frequently rely on information, structure, and multifunctionality, whereas engineering solutions are constrained by available materials, processes, and production resources [1,2,3]. The success of biomimetic innovation therefore depends not only on the originality of the biological analogy but also on the traceability of the transfer and its industrial feasibility.

Industry 4.0 provides a broad technical toolset for this transfer. Artificial intelligence and large-scale data resources support the search and association of biological patterns; parametric and generative design and topology optimization translate those patterns into digital geometries. Simulation and digital twins can support pre-production validation, additive manufacturing can realize complex geometries, and sensors and industrial IoT can monitor function and provide feedback. The use of a digital technology, however, does not make a solution inherently scalable. Process capability, cost, quality stability, standardization, and life-cycle evidence frequently lag behind design performance [4,5,6,7,8].

Previous reviews and application studies have established that biomimetic principles can be translated into complex geometries, optimized material distributions, multiscale surfaces, and additively manufactured prototypes, and that digital tools can improve search, design, simulation, and testing [4,9,10,11,12,13,14]. What remains unresolved is the transition from a successful specimen or prototype to a repeatable production system: the literature does not yet explain consistently how multiple technologies are integrated, how the critical biological function is preserved under process variation, or how quality, rate, total cost, standards, field performance, and life-cycle value change with scale.

### Research Gap in Relation to Recent Reviews

Recent studies address separate parts of the digital biomimetics process. Gralow et al. [4] examined the compatibility of biomimetic design methods with laser additive manufacturing and discussed the role of digitalization. Du Plessis et al. [9] reviewed geometric and functional opportunities in three-dimensional concrete printing. Shen et al. [13] traced a systematic route from a biological mechanism to a multiscale drainage surface, and Shu et al. [14] combined deep-learning-designed silicifying peptides with biomimetic nanosilica synthesis and anti-UV validation. These studies demonstrate technical feasibility in particular design, material, and process settings, but they do not provide a production-route-neutral account of industrial repeatability and scale.

A gap nevertheless remains at the production-system level. Existing work rarely examines how several Industry 4.0 technologies operate together while also accounting for process capability, repeatable quality, production rate, economics, industrial maturity, and field or life-cycle evidence. The relevant unit of analysis is the full technology–process–evidence chain from the biological model to sustained operational performance, not the novelty of a single geometry or prototype. Table 1 positions the review against the most closely related studies and clarifies its distinctive production-system emphasis.

The principal research question is: How do Industry 4.0 technologies enable the development, validation, manufacture, and industrial-scale application of biomimetic designs? The question is addressed through four dimensions: (i) the functions of digital technologies within the transformation chain; (ii) evidence of manufacturability and scalability; (iii) operational and sustainability performance; and (iv) the technical and managerial barriers to moving from laboratory settings to industrial scale.

The distinctive contribution is to treat biomimetic design as a production-management problem rather than as a descriptive catalog of biological examples. The framework integrates the chain biological model → biomimetic design principle → Industry 4.0 technology → manufacturing method → manufacturability and scalability → operational and sustainability performance. Industry 5.0 does not replace the technical focus; it is used as a higher-level governance perspective on human-centricity, sustainability, and resilience.

## 2. Conceptual Background

### 2.1. Biomimetic Transfer as a Production-Management Problem

Biomimetic transfer may begin with an engineering problem that prompts a search for biological analogies or with a biological phenomenon whose principle is translated into a technical application. Both routes require disciplined abstraction: direct copying, weak analogical alignment, and premature fixation are recurrent sources of design error [1,2,3]. Transferring a biological example into a technical design consequently requires three forms of fidelity. Biological fidelity concerns the correct interpretation of the structure–function relationship in the source system; technical fidelity concerns its translation into computable requirements; and manufacturing fidelity concerns reproducibility within material, process, and tolerance limits. If any of these levels is missing, a design may appear biomimetic without generating industrial value. Terminological precision is also necessary because biomimetic, bio-inspired, and biomorphic developments do not imply equivalent transfer depth or sustainability outcomes [15,16,17]. Extending biological transformation into manufacturing systems therefore requires organizational, process, and life-cycle management as well as technology [6,7,8].

### 2.2. Enabling Functions of Industry 4.0 Technologies

Industry 4.0 technologies enter the biomimetic transformation process at different stages and serve different purposes. Table 2 distinguishes AI, machine learning, generative AI, large language models, generative and parametric engineering design, additive and digital manufacturing, digital twins, sensing, and robotics. It then maps the direct Group A evidence onto E0–E5. Reviews and other contextual sources are used to explain technological roles but are not assigned an E-level, because the scale applies to evidence reported for specific cases.

The mapping reveals both a division of technological roles and a clear evidence gap. AI is used as the broad field; machine learning covers prediction, classification, surrogate modeling, and anomaly monitoring; generative AI produces candidate representations; LLMs support language-mediated retrieval and decisions; and engineering generative design searches a constrained design space. The last of these should not be confused with generative AI. Digital twins also differ from static simulation because they require model updates from process or field data. No direct case reached E4 or E5, so pilot and serial-production evidence is absent from the selected set.

### 2.3. Manufacturability and Scalability

Manufacturability extends beyond producing a geometry once and is production-route neutral. It requires a qualified material–process combination, repeatability, dimensional tolerance, controlled defect probability, residual-stress management where relevant, fatigue or durability evidence, post-processing time, production rate, yield, and an executable quality-control plan. Additive manufacturing is one possible final-production, tooling, or prototyping route; molding, machining, forming, surface processing, assembly, and hybrid routes may be more suitable at other volumes. Industrial scalability requires the critical function and conformance to be sustained across volume, site, and time, while CAPEX, OPEX, cycle time, labor, tooling, yield loss, post-processing, maintenance, unit cost, supply continuity, standards, and life-cycle performance remain acceptable [5,40,42,43].

### 2.4. Positioning Industry 5.0

Industry 5.0 is treated not as a separate technology package that replaces Industry 4.0, but as a framework that directs technology choices toward specific values. This positioning follows the European Commission’s formulation of a sustainable, human-centric, and resilient industry [44]. Human-centricity concerns safety, ergonomics, explainable decision support, and human–machine collaboration; sustainability concerns energy, materials, waste, and life-cycle impacts; and resilience concerns adaptability, modularity, self-monitoring, and recovery from disruption [45,46,47].

## 3. Materials and Methods

### 3.1. Review Design

This research was designed as a critical integrative review rather than as a bibliometric performance analysis or a systematic review claiming exhaustive record screening. Its purpose is to compare heterogeneous technical evidence under common concepts and to explain mechanisms through which biomimetic solutions move from biological knowledge to production and industrial outcomes. A purposive, maximum-variation full-text evidence set was therefore assembled to represent different technologies, application domains, transformation stages, and evidence types. The review does not estimate technology prevalence or claim comprehensive coverage; its interpretive claims are bounded to the 50 selected full texts. The synthesis follows the integrative and conceptual review principles discussed by Snyder [48] and Jaakkola [49].

### 3.2. Web of Science Search and Construction of the Evidence Set

The Web of Science Core Collection was searched on 3 August 2026 using a broad discovery strategy organized into fourteen concept families: core biomimetic manufacturing; artificial intelligence and biological knowledge; generative, parametric, and topology design; CAD/CAE/FEA/CFD and simulation; digital twins, cyber–physical systems, and digital threads; additive manufacturing; materials, lattices, and surface engineering; sensors, robotics, and adaptive control; manufacturability and scaling; operational performance; sustainable manufacturing; Industry 4.0; Industry 5.0; and reviews/frameworks. The exported WoS records were retained in Excel files and manually screened within each query family. Screening began from relevance-ranked results and assessed title, abstract, and keyword fit; highly cited foundational records were identified through Times Cited; and recent records published in 2023–2026 identified through Publication Date were also examined to balance conceptual influence and currency. The discovery pool supported topic coverage rather than a systematic prevalence estimate. The exact Smart Search Boolean strings and a record-level log assigning every excluded record to a screening reason were not retained; therefore, exact stepwise exclusion counts from the broad pool to the acquired PDFs are not reported. Table 3 and Supplementary File S1 document the preserved selection procedure and all auditable downstream counts. Appendix A provides a concise summary of the search and evidence-set construction.

Gold Open Access, Article/Review Article, and English-language filters were applied at the discovery and acquisition stage. Gold Open Access was used as an operational full-text auditability boundary: every coded mechanistic and quantitative claim had to be checked against the complete article and remain accessible for revision audit. It was not used as a proxy for scientific quality, and the restriction does not imply that non-Gold publications are less influential. The boundary may exclude important subscription-based work in manufacturing, materials, robotics, design, and mechanical engineering and is therefore reported as a coverage limitation.

Within each query family, exported records were screened manually in relevance order using title, abstract, and keyword fit. Highly cited foundational papers and recent papers published in 2023–2026 were also examined. A full text was eligible only when it identified a biological source or principle, linked it to a traceable technical solution, and provided verifiable evidence concerning design, validation, manufacturing, monitoring, performance, manufacturability, or scale. Eligible papers were then selected purposively to cover the seven transformation stages, complementary Industry 4.0 technology families, different application areas, and different forms of evidence. The procedure was neither random nor exhaustive. All claims considered for coding were checked in the complete article. This process produced 52 acquired PDFs; file-level quality control identified two duplicate copies, leaving 50 unique full texts. The final set comprises 12 direct Group A cases, 25 Group B technology or process-depth studies, and 13 Group C studies dealing with production management, life cycle, scaling, or Industry 5.0. Table 3 reports the preserved procedure and the downstream counts. It does not reconstruct intermediate exclusion totals because a record-level exclusion log was not retained.

A currency and originality check was conducted on 3 August 2026 against recent adjacent studies. Shen et al. [13], Shu et al. [14], and Zeng et al. [50] were used only to clarify the research gap and positioning; they were not added to the 50-study coding set, technology-pattern synthesis, or evidence-level assessment. Foundational design-process, terminology, sustainability, and Industry 5.0 sources [1,2,3,15,44] were likewise used to strengthen conceptual interpretation rather than as coded application evidence.

### 3.3. Eligibility, Coding, and Evidence Assessment

Eligibility was assessed according to whether a publication provided a traceable connection between a biological principle, a technical solution, and at least one production-relevant form of evidence. Table 4 summarizes the access, publication type, language, content, and evidence rules used to distinguish codable manufacturing evidence from studies employing bio-inspired terminology without an explicit production relationship.

The eligibility rules establish the minimum evidentiary threshold, but inclusion alone does not make heterogeneous studies directly comparable. The next analytical step therefore decomposed each eligible publication into the same sequence of biological source, design principle, digital technology, manufacturing route, scale, performance, and limitation. This common structure allowed technical demonstrations and production-relevant evidence to be compared without treating different application sectors as methodologically equivalent.

The coding scheme was organized around the complete transformation chain and expanded to distinguish material, manufacturing route, manufacturability, production scale/economics, validation, and measurable Industry 5.0 indicators. Table 5 defines the analytical dimensions used to separate a one-off technical demonstration from repeatable and economically scalable production.

Analytical coverage and evidentiary strength were assessed separately. Table 6 rates the evidence supporting a particular published claim; it does not rate the maturity of a whole technology or organization. Each level has a minimum observable gate and an exclusion boundary. The distinction is especially important for E4 and E5, because evidence from a finite pilot is not sufficient for a serial or commercial classification.

Table 5 and Table 6 answer different questions. Table 5 defines what was coded, whereas E0–E5 records what a publication actually demonstrates. E4 requires an integrated pilot in a stated relevant environment, predefined acceptance criteria, repeated runs or batches, quantified conformance or variation, and a reported scale and duration. E5 requires routine serial production or sustained field or commercial operation over multiple batches, deployments, or periods, together with evidence on quality stability or process capability, production economics, and, where relevant, qualification and maintenance. A technically successful but finite and uncosted pilot therefore remains E4. TRL addresses technology or system readiness, while MRL addresses manufacturing capability and risk [51,52]. Neither is a scale for rating the strength of a published claim, and no direct conversion to E0–E5 is proposed.

### 3.4. Reliability of the Synthesis and Rules of Interpretation

Coding proceeded in two stages. Bibliographic fields and analytical roles were first consolidated; technology, production, performance, and limitation claims were then verified against the full texts. The coding unit was not the paper as a whole but the explicitly reported biological model–technology–production–performance connection. Quantitative results were retained only when the comparison basis, conditions, and units were identifiable; missing replication, dispersion, confidence intervals, or inferential tests were recorded as not reported rather than inferred. “Potential,” “proposed,” “demonstrated in simulation,” “experimentally validated,” and “implemented in a relevant environment” were not treated as equivalent. Direct Group A claims were assessed on the E0–E5 scale, while technology reviews and contextual management sources were kept analytically separate.

The review relies on single-researcher coding; no independent second coder or inter-rater reliability coefficient was available. Interpretive risk was reduced by linking each principal finding to a source–claim trail, rechecking quantitative results in the full text, assigning the lower evidence level in ambiguous cases, and excluding contextual publications from counts of application evidence. Technology frequencies were not reported as prevalence estimates; the synthesis concerns mechanism patterns and evidence levels within the selected 50 full texts.

## 4. Results

### 4.1. Evidence Structure and the Technology–Application Distinction

The 50 full texts were organized by analytical function: Group A comprises 12 direct mechanism/application studies extending from biological principles to production and performance; Group B comprises 25 studies providing technology and process depth in AI, digital twins, sensors, robotics, additive manufacturing, materials, and surface engineering; and Group C comprises 13 studies addressing life cycle, smart manufacturing, scale-up, operational monitoring, and Industry 5.0. These counts reconcile the manuscript with Supplementary File S1. The groups describe analytical roles, not the quantitative distribution of the field, and contextual studies are not interpreted as direct biomimetic application evidence.

Across the evidence, biomimetic production is enabled not by a single technology but by a sequential digital chain. AI and knowledge models add value in search and abstraction; parametric design and optimization in geometry generation; CAE in virtual validation; additive manufacturing in physical realization; and sensors, control, and IoT in operational feedback. Digital twins could establish data continuity across these stages, but evidence within the selected biomimetic set is limited and mainly conceptual [12].

### 4.2. Artificial Intelligence: From Biological Knowledge to the Design Space

AI-related terms refer to different functions and are not used interchangeably. AI is the umbrella for computational knowledge and decision systems. Machine learning learns predictive or classificatory relationships, supplies surrogate models for design-space search, and detects anomalies [21,22]. Generative AI produces new candidate content or representations [11,23,24], whereas engineering generative design and topology optimization search a parameterized, constraint-defined geometry space [26,27,29]. LLMs provide language-mediated retrieval, explanation, and dialog around biological knowledge [25]; they do not independently validate a design. Process monitoring and closed-loop decisions require sensor data, explicit control logic, and human authority rather than an AI label alone.

Industrial use remains constrained by data structure and validation. Datasets rarely combine biological morphology, material/process variables, tolerance and defect data, cost, and life-cycle outcomes for the same case. External validation, uncertainty, explainability, cybersecurity, and human review are required when predictions influence design release, process control, or safety-critical decisions [18,19,20].

### 4.3. Generative Design, Topology Optimization, and Digital Validation

Generative design and topology optimization represent biological principles as variable design rules rather than fixed shapes. Parametric optimization of L-system lightweight structures generated designs adapted to different load cases, with approximately 74–84% mass reductions relative to the initial geometry [26]. Gradient cellular solids linking additive manufacturing with coupled mechanoelectrical response, biomimetic microchannel optimization, the interlocking geometry of walnut-shell cells, and Voronoi lattices derived from sea-urchin stereom show that biological relations can be modeled at the geometry–material–load level [28,53,54,55]. Nature-inspired topology optimization of metal 3D-printed joints likewise demonstrates that performance objectives must be defined jointly with production routes and constraints [27,56].

Digital validation reduces pre-production risk, but its value depends on alignment between model and process. Generative design reduced support material by more than 40% in one additive-manufacturing study, which also identified computational cost and the need for low-cost process simulation as major barriers [29]. Optimization detached from production economics therefore provides no assurance of scalability.

### 4.4. Additive Manufacturing, Advanced Materials, and Surface Engineering

Additive manufacturing occupies a central position in the selected evidence because it can realize hierarchical lattices, curved microchannels, multi-material structures, and internal geometries. It is not, however, synonymous with industrial scalability. AM may be the final route, a bridge to tooling or molding, or only a prototyping method; route selection must be based on required volume, tolerance, rate, yield, post-processing, qualification, and cost. Biomimetic surfaces and components may also depend on laser processing, molding, assembly, machining, forming, or hybrid routes [57,58,59,60,61]. Across routes, build/process orientation, anisotropy, defects, residual stress, surface quality, fatigue, and quality control can substantially alter function [10,43,61].

Correctly linking biological structure to function can yield measurable benefits. A trabecular-bone-derived mass-distribution strategy increased bending stiffness by 66% and 111% relative to equal-mass designs [30]. A fish-scale pattern transferred to aluminum by laser ablation achieved a maximum drag reduction of 10.26% in closed-water-tunnel tests [32]. In both cases, value arose from a functional mechanism realized through manufacturing rather than from visual similarity.

Eco-efficient supports and bio-inspired infill geometries for 3D-printed concrete envelopes illustrate how material or energy benefits can be reported against an explicit baseline [31,33]. Whether such benefits persist across production volumes and the full life cycle requires separate verification.

### 4.5. Robotics, Sensors, the Industrial Internet of Things (IIoT), and Operational Feedback

Biomimetic robotics adds sensing and control to the design–production chain. A modular soft actuator inspired by earthworm locomotion combined 3D-printed molds and covers, silicone molding, an embedded flexible sensor, and PID control. Production took less than one day, and durability was tested over 14,752 actuation cycles [35]. Plant-inspired sensing and actuation similarly show that material behavior and control must be co-designed [40,41].

Sensors and analytics allow a function to be monitored in use. A fully 3D-printed tactile sensor combined human-skin-inspired interlocking papillae with an auxetic structure, achieving normal and shear sensitivities of 0.63 kPa^−1^ and 0.92 N^−1^, respectively, and a stable response over 1500 loading cycles [34]. Machine-learning classification of wind from wing deformation shows that the structure itself can become a sensing medium [62].

At the production-system level, an IoT architecture used vibration sensors and a real-time autoencoder to monitor motor anomalies on a prototype conveyor. Network latency and control integration were identified as future scaling barriers [22]. Distributed cyber–physical production control and smart-manufacturing-inspired scale-up approaches emphasize self-awareness, data feedback, and modular experimentation [36,37]. Because these sources do not provide direct biomimetic application performance, they are used only as contextual evidence for closed-loop scaling logic.

### 4.6. Verified Technology–Production–Performance Relationships

Table 7 includes the complete Group A subset (all 12 direct mechanism/application studies), not a selectively chosen sample. To meet the requested fields without duplicating the same information, Part (a) reports biological model/property, material, manufacturing route, digital technology, and production scale; part (b) reports baseline/control, quantitative metric, validation conditions, statistical treatment, E-level, and major limitation. Together they provide the requested source-to-evidence audit trail.

The comparison is mechanism-based rather than sector-based. Inclusion of all eligible Group A cases prevents post hoc selection of only high-performing examples. The aim is not to rank applications or infer average effects, but to test whether a technically successful biomimetic transfer is accompanied by evidence of repeatability, production scale, and operational feasibility. Industrial enablement requires both realization of the intended biological function and evidence that the function can be maintained under manufacturing and deployment conditions.

Part (a) of Table 7 establishes the mechanism, material/process route, and demonstrated scale for every direct case. Part (b) then changes the unit of comparison to the reported outcome: it identifies the baseline, exact metric, validation method, statistical treatment, evidence level, and limitation. Distributing the requested fields across two companion tables removes the earlier overlap while preserving readability.

Read together, parts (a) and (b) of Table 7 reveal an asymmetry between technical achievement and industrial evidence. Biological functions are translated into identifiable materials, geometries, and component-level gains, but repeatability, dimensional tolerance, defect probability, residual stress, fatigue, production rate, yield, post-processing time, and formal quality control are usually unreported. Evidence becomes thinner still for multi-batch production, total cost, certification, and field operation. Missing values are recorded as not reported rather than inferred.

### 4.7. Bottlenecks in Manufacturability and Scalability

Industrial scaling cannot be attributed to additive manufacturing or to a single missing technology. Eight recurrent, production-route-neutral bottlenecks correspond to Table 8: biological abstraction; data/AI; computation and digital validation; manufacturing process capability; quality and standards; production economics; sustainability; and operational deployment. The expanded process-capability category includes repeatability, dimensional tolerance, defect probability, residual stress, fatigue, post-processing time, production rate, yield, and quality control. The economics category includes CAPEX, OPEX, labor, cycle time, tooling, yield loss, post-processing, maintenance, and unit cost.

The bottlenecks interact. A biologically valid mechanism may fail during manufacturing validation, a stable process may still be commercially infeasible, and a cost-effective component may be unsuitable for deployment when maintenance, cyber–physical security, monitoring, or regulatory requirements remain unresolved. Industrial scalability therefore requires the critical function, conformance, production rate, and acceptable economics to be maintained across volume, site, and time. It is not achieved merely by enlarging or repeating an additive-manufacturing build.

### 4.8. Consolidated Quantitative Findings

Table 9 brings together the quantitative findings reported in the 12 direct cases and groups them by outcome domain. Effect sizes are not pooled because the studies differ in baselines, units, materials, validation methods, sample sizes, and E-levels. The table is a comparative summary, not a meta-analysis.

## 5. Discussion

### 5.1. How Industry 4.0 Enables Biomimetic Design

The evidence points to a combination of technologies rather than a single enabling tool or production route. Industry 4.0 makes biological knowledge searchable and computable, supports design-space exploration through machine learning, generative or parametric design, topology optimization, and simulation, enables geometry realization through qualified manufacturing routes, and permits function to be measured through sensors, IIoT, robotics, and control. As Table 2 shows, the direct evidence is concentrated at E1–E3. Digital twins and fully closed-loop integration are discussed mainly in contextual sources and are not demonstrated directly at E4–E5.

These technologies are complementary components of one production chain rather than substitutes. AI or topology optimization detached from manufacturing constraints can generate high-performing but unprintable geometries. CAE reliability is uncertain without experimental calibration, while a digital twin without sensors and feedback remains a static virtual representation. Enabling value depends less on the number of technologies than on a traceable connection among biological source, design variable, process parameter, and performance data.

### 5.2. Distinguishing Technological Intensity from Industrial Maturity

Technical performance is generally validated for a specific specimen, load case, or controlled laboratory setting. Evidence for serial-production volume, process capability, supply continuity, standards compliance, and field maintenance is more limited. High mechanical performance alone does not establish industrial maturity. From a production-management perspective, the unit of assessment is the socio-technical production system comprising design data, process route, quality plan, monitoring infrastructure, and life-cycle economics.

The selected studies provide E1–E3 evidence of technical feasibility. E4 would require a stated relevant environment, predefined acceptance criteria, repeated runs or batches, quantified conformance or variation, and a reported scale and duration. E5 would additionally require sustained routine production or field operation, stable quality, economic evidence, and qualification or maintenance data. None of the direct cases met those E4–E5 requirements across the complete chain. The pressing need is therefore for transition studies that test whether function is preserved under realistic process variation and sustained operation, rather than for further one-off prototypes alone.

### 5.3. Implications for Production Management

Organizations should manage biomimetic projects as portfolios of staged evidence rather than as technology-acquisition projects. After biological abstraction, teams should validate model–experiment agreement, route-specific process windows, repeatability, tolerance, defect and residual-stress control, fatigue/durability, post-processing, production rate, yield, and acceptance criteria. A digital thread should trace design decisions back to the biological source and forward to quality and field outcomes; AI recommendations should remain auditable decision support that responsible engineers can explain, challenge, and override.

Production economics should be evaluated from the first route decision rather than after technical optimization. The required volume and service value determine whether AM is a final route, a tooling/prototyping route, or should give way to molding, machining, forming, or a hybrid process. The business case should report CAPEX and capacity assumptions; OPEX for energy, material, labor, and consumables; cycle time and production rate; tooling; first-pass yield, scrap and rework; post-processing; maintenance and downtime; and unit cost across volume scenarios. In the selected evidence, [33] provides bounded material, time, energy, and cost comparisons, and [35] reports approximately 22 h of manufacture/assembly, but enterprise-level volume-sensitive economics are generally not reported. Values marked NR are not inferred.

### 5.4. Interpretation Through Industry 5.0

Industry 5.0 is used here as an evaluative perspective, not as an empirical result attributed to every selected study. Table 10 expresses human oversight, worker safety, energy, material efficiency, life-cycle impact, resilience and adaptability, and cybersecurity as measurable indicators and decision rules. These indicators are intended for future pilot and field studies. When a paper reports only technical potential, no Industry 5.0 outcome is claimed.

Claims about Industry 5.0 require measured outcomes. A biology-derived label does not in itself demonstrate sustainability [15,50], and a reduction in mass must be considered together with energy use, scrap, supports, rework, post-processing, service life, and end-of-life. Likewise, a soft or adaptive robot cannot be described as human-centric without examining operator authority, workload, exposure, safe-stop performance, and incident risk. Connected sensing and digital twins introduce additional data integrity and cyber–physical risks. In this review, Industry 5.0 therefore provides criteria for evaluating evidence rather than an outcome automatically assigned to the selected cases [44,45,46,64].

### 5.5. Evidence Hierarchy for Sustainability Claims

Sustainability is often discussed through lightweighting or reduced support material. These indicators are relevant, but net environmental value requires comparison of raw materials, production energy, failed builds, post-processing, service life, maintenance, and end-of-life scenarios on the same functional unit. Prior biomimetics research cautions that biological derivation and technical efficiency should not be treated as proof of sustainability without explicit evaluative criteria [15,50]. Material reduction alone is therefore a hypothesis to be tested through comparative life-cycle assessment and life-cycle engineering [63]. The same distinction applies to resilience: modular or adaptive design indicates only potential unless recovery time and continuity of service are measured.

## 6. Technology–Production–Performance Transformation Framework

**Figure 1:**
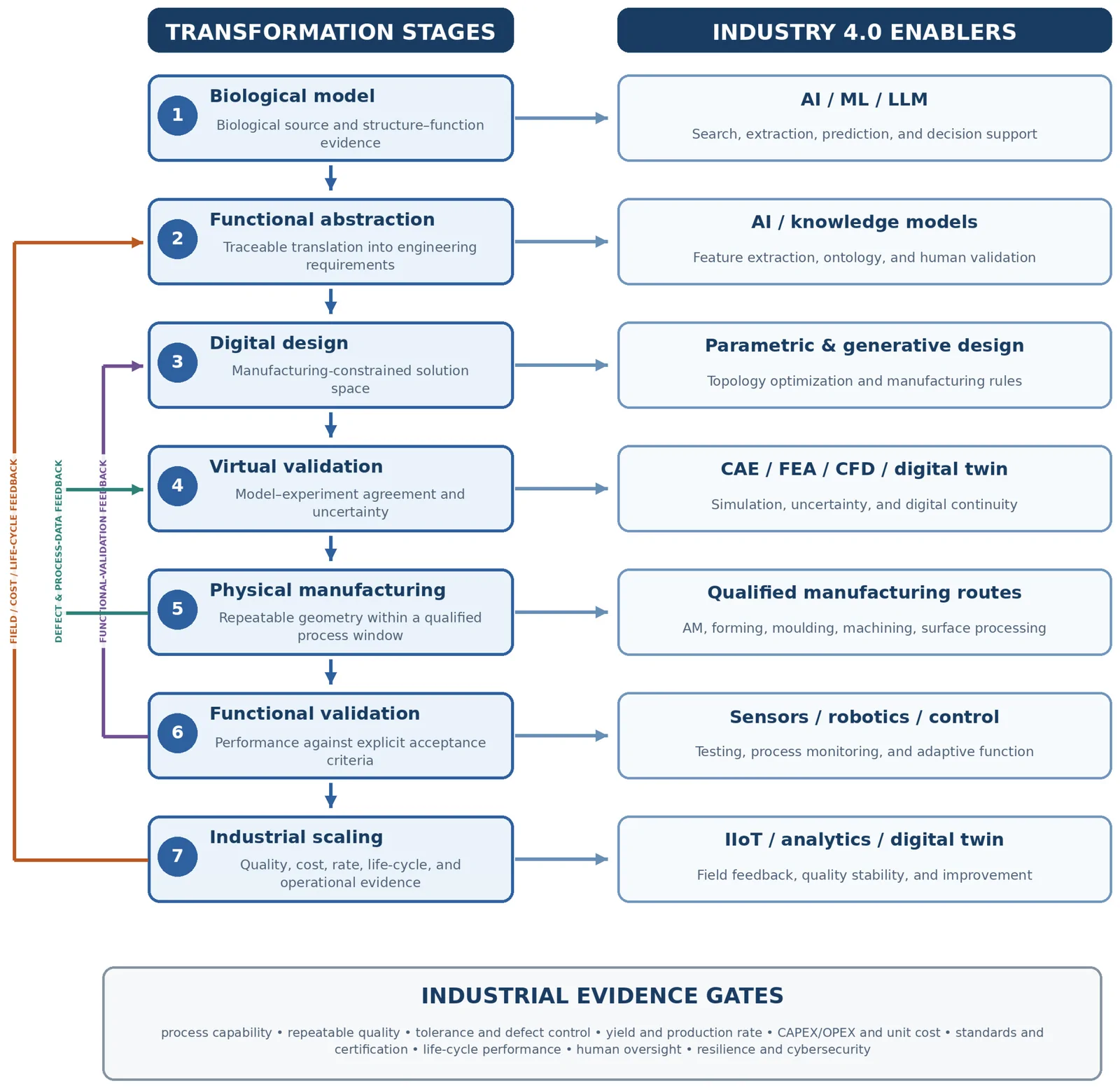
Author-derived technology–production–performance framework with explicit validation, manufacturing/process-monitoring, and field-performance feedback loops. Note: The framework is a conceptual synthesis grounded in the coded 50-study evidence. It is not a validated maturity scale. The feedback loops show how functional deviations, manufacturing/process data, and field/economic/life-cycle evidence revise earlier design and optimization decisions; the complete study-stage matrix is provided in Supplementary File S1. Blue arrows indicate forward progression and enabling links; orange, green, and purple arrows indicate field/life-cycle, manufacturing/process-data, and functional-validation feedback, respectively.

The seven-stage framework was developed by organizing the common coding chain used for all 50 full texts. The empirical material remains the study-level technologies, materials and processes, baselines, measured outcomes, E-levels, and limitations reported in Section 4.1, Section 4.2, Section 4.3, Section 4.4, Section 4.5, Section 4.6, Section 4.7 and Section 4.8 and Supplementary File S1. The framework arranges that material into seven stages: biological model, functional abstraction, digital design, virtual validation, physical manufacturing, functional validation, and industrial scaling. It should not be read as an empirically validated maturity model. Table 11 gives the references supporting each stage, and Supplementary File S1 provides the full 50-study by seven-stage matrix. Figure 1 also shows three feedback paths: functional deviations return to design and optimization; manufacturing defects and process-monitoring data return to virtual validation and design; and field performance, cost, maintenance, and life-cycle evidence inform earlier requirements and design decisions.

### Propositions Derived from the Synthesis

Five propositions were developed from mechanisms that recur across the reviewed full texts. They are intended as testable statements, not as effect estimates or statistically tested causal claims. Table 12 identifies the direct studies and E-levels associated with each proposition and lists contextual support separately. The fuller proposition–evidence map and its inferential boundaries are provided in Supplementary File S1.

The propositions place the emphasis on how technologies are integrated rather than on whether advanced technologies are simply present. They extend beyond the selected evidence and should be tested in subsequent research. Direct support ranges from E1 to E3; none is supported by E4–E5 evidence across the complete chain. Multiple-case, pilot-production, and field studies are needed to determine whether stronger integration across stages improves repeatability, reduces scale-up risk, and produces measurable Industry 5.0 outcomes.

## 7. Theoretical and Practical Contributions

### 7.1. Theoretical Contribution

The main theoretical contribution is to separate the findings reported by individual studies from the transformation framework developed through the review. This shifts the unit of analysis from an isolated biological example to a production-route-neutral technology–process–performance system. It also distinguishes technical performance from industrial readiness through explicit evidence gates and treats digital continuity and manufacturing constraints as mechanisms to be examined empirically. Industry 5.0 is handled in the same way: as a set of measurable evaluation criteria, not as an outcome that follows automatically from biomimetic design.

### 7.2. Methodological Contribution

The methodological contribution is to code a bounded, purposively selected set of accessible Web of Science full texts through evidence of biological models, technologies, manufacturing methods, performance, and scale rather than through title matching alone. The A–B–C analytical groups separate direct applications from technology/process depth and production-management context, while E0–E5 distinguishes claim strength without conflating it with TRL or MRL. The study-stage and proposition-evidence matrices make the derivation of the framework auditable at the reference level.

### 7.3. Contribution to Production and Operations Management

The framework clarifies which evidence is required at each decision stage. Design teams can integrate AI and optimization with material/process constraints; manufacturing engineers can quantify repeatability, dimensional tolerance, defects, residual stress, fatigue, post-processing time, production rate, yield, and quality-control capability; and operations managers can evaluate CAPEX/OPEX, labor, tooling, unit cost, maintenance, supply, standards, field reliability, and life-cycle indicators at pilot and scaling gates. This makes visible the gap between a prototype produced once and a function delivered consistently and economically.

### 7.4. Managerial and Policy Implications

Managers should not base investment decisions solely on mechanical performance or geometric complexity. Project gates should jointly require a traceable biological mechanism, model–experiment agreement, process capability, unit and total cost, standards compliance, and life-cycle impact. Public support and technology programs could emphasize pilot-environment validation, open data and ontology infrastructure, standardized test methods, and clear human accountability for AI-supported decisions rather than the number of prototypes alone.

## 8. Limitations and Future Research

The review is limited to the Web of Science Core Collection, English-language Articles/Reviews, the Gold Open Access filter, and a purposively selected full-text set. Gold OA improved complete-text auditability but may exclude influential subscription-based research in manufacturing, materials, robotics, design, and mechanical engineering. The preserved Excel exports document the structured manual selection procedure but do not contain the exact Smart Search Boolean strings or a record-level exclusion log linking every screened record to an exclusion reason. Accordingly, exact stepwise counts from the broad discovery pool to full-text acquisition are unavailable and are not reconstructed. The 50 publications were selected through relevance screening, foundational and recent-literature coverage, full-text eligibility, and maximum analytical variation rather than exhaustive title-and-abstract screening or random sampling. Consequently, the findings explain mechanisms within the selected evidence set and do not estimate literature size, technology prevalence, or average effects. Single-researcher coding introduces interpretive risk, and heterogeneity of metrics, materials, and scales precludes meta-analytic effect-size estimation. Table 13 translates these limitations into a focused future research agenda.

The agenda prioritizes transition evidence over the production of additional isolated prototypes. The most consequential studies will connect laboratory performance to pilot-production data, process-capability measures, total-cost evidence, life-cycle comparison, and operator- or field-level outcomes. Such designs would allow the proposed decision gates to be refined and would show which combinations of digital technologies actually reduce the gap between biomimetic novelty and industrial deployment.

## 9. Conclusions

Biomimetic design predates Industry 4.0, but Industry 4.0 supplies much of the infrastructure needed to turn biological knowledge into engineering solutions that can be manufactured, verified, and potentially scaled. Within that infrastructure, machine learning supports prediction, optimization, and monitoring; generative AI and LLMs assist candidate generation and knowledge interaction; engineering generative design and topology optimization explore constrained geometries; CAE and digital twins support validation and continuity; qualified manufacturing routes realize the intended function; and sensors, robotics, and IIoT provide feedback.

The 50 selected full texts report measurable gains in material and support reduction, stiffness, drag, energy performance, sensor placement, and durability under specified study conditions. These findings cannot be generalized into prevalence or average-effect claims beyond the purposive evidence set. Technical performance is also insufficient for industrial value: evidence concerning process stability, repeatable quality, total cost, standardization, certification, life cycle, and operational integration is markedly weaker.

The critical bottleneck is not whether a complex geometry can be produced once, but whether the same function can be reproduced through a controlled process, consistent quality, and acceptable cost. The proposed technology–production–performance framework explains this transition through seven evidence gates, from biological model to scaling decision. Industry 5.0 provides the governance framework that directs the technical infrastructure toward human-centric, sustainable, and resilient outcomes.

## Figures and Tables

**Table 1:** Analytical positioning of this review relative to closely related studies.

Study	Primary Focus	Main Contribution	Distinctive Emphasis of This Review
Gralow et al. [4]	Biomimetic design and laser additive manufacturing	Relationship between the digitalization of design methods and the manufacture of complex geometries	Technology orchestration beyond a single process; process capability and scaling evidence
du Plessis et al. [9]	Biomimicry in 3D concrete printing	Applications and research agenda for structural and geometric biomimicry	Production–performance–maturity comparison across materials and applications
Shen et al. [13]	Systematic biomimicry and multiscale drainage surfaces	Workflow from a pitcher-plant mechanism to rapid construction and controlled-drainage validation	Links multiple Industry 4.0 technologies to manufacturability, quality, cost, and scale decisions
Shu et al. [14]	AI-guided biological knowledge and materials synthesis	Deep-learning-designed silicifying peptides with biomimetic nanosilica and anti-UV validation	Positions AI within a broader manufacturing technology chain and evaluates industrial evidence
This review	Industrialization of biomimetic design through Industry 4.0	Technology–production–performance framework from biological model to production and performance	Joint assessment of manufacturability, scalability, industrial maturity, and Industry 5.0 governance

Note: Shen et al. [13] and Shu et al. [14] were identified through a currency and originality check conducted outside the primary coding set.

**Table 2:** Distinct technology roles and direct application evidence across E0–E5.

Technology Class	Distinct Role in the Transformation Chain	E0	E1	E2	E3	E4	E5
AI/knowledge models	Biological search, feature extraction, ontology building, and expert-supported abstraction; contextual support [18,19,20]	—	—	—	—	—	—
Machine learning	Prediction, classification, surrogate modeling, optimization, and anomaly monitoring	—	—	[21]	[22]	—	—
Generative AI	Generation of candidate concepts or material/design representations; distinct from constraint-based engineering optimization; contextual support [11,23,24]	—	—	—	—	—	—
Large language models	Language-mediated retrieval, explanation, and decision support for biological knowledge; contextual support [25]	—	—	—	—	—	—
Parametric/generative design/topology optimization	Manufacturing-constrained geometry generation and load–material–shape optimization	—	[26,27]	[28,29,30]	—	—	—
Additive and digital manufacturing	Physical realization of complex, hierarchical, multi-material, or surface geometries	—	[26,27,31]	[21,28,29,30,32,33,34]	[35]	—	—
Digital twins	Model updating and continuity among design, process, and field data; contextual support [12,36,37,38]	—	—	—	—	—	—
Sensing/IIoT	Functional measurement, anomaly detection, process monitoring, and field feedback	—	—	[34]	[22,35]	—	—
Robotics/control	Integrated adaptive function, motion, human–machine interaction, and closed-loop control; contextual support [39,40,41]	—	—	—	[35]	—	—

Note: Entries in E0–E5 are limited to direct Group A claims. A dash indicates that no direct case at that evidence level was identified in the selected set; it does not imply that the technology cannot reach that level.

**Table 3:** Auditable selection flow from broad discovery to the final purposive evidence set.

Selection Stage	Auditable Record	Decision Rule and Boundary
1. Broad discovery	WoS Core Collection; 14 concept families; searched 3 August 2026	Biological anchors were paired with technology, manufacturing, performance, and industrialization concepts. This was a discovery universe, not a single PRISMA sampling frame.
2. Operational filters	Gold Open Access; Article/Review Article; English	Complete-text auditability and consistent publication/language scope. Gold OA was not treated as a quality criterion.
3. Relevance check	Manual WoS Excel screening; record-level exclusion log not retained	Within each query family, relevance-ranked records were assessed by title, abstract, and keywords; highly cited foundational and recent (2023–2026) records were also examined.
4. Full-text eligibility	Full article inspected before coding	Required an explicit biological source/principle, a traceable technical solution, and verifiable production-relevant evidence.
5. Maximum-variation selection	Non-random purposive selection	Coverage sought across all seven stages, complementary technology families, application domains, and evidence types; no field-prevalence estimate was intended.
6. Acquisition and file QC	52 acquired PDFs	Two duplicate file copies were removed; no unique study was excluded at this step.
7. Final analytical set	*n* = 50: Group A = 12; Group B = 25; Group C = 13	All 12 direct mechanism/application cases; 25 technology/process-depth studies; 13 production-management, life-cycle, scaling, and Industry 5.0 contextual studies.

**Table 4:** Inclusion and exclusion criteria.

Inclusion	Exclusion
Gold Open Access full text available for complete claim verification	Subscription-only or inaccessible record whose methods/results could not be audited
English-language Article or Review Article	Other publication type or language outside the operational boundary
Explicit link between a biological model/principle and a technical solution	Optimization algorithm labeled only as ‘bio-inspired’ without a biological transfer mechanism
At least one digital design, validation, manufacturing, monitoring, or control technology	Computing study without a physical design/manufacturing relationship
Manufacturing method, prototype, experiment, process, performance, or industrial implication	Clinical/biomedical study that cannot be coded from a manufacturing perspective
Evidence on manufacturability, performance, scaling, or barriers	Only a general sustainability claim without a traceable production relationship

**Table 5:** Full-text coding dimensions, including manufacturability and production economics.

Dimension	Coding Question
Biological source/model	Which organism, tissue, structure, motion, or system principle is used, and what structure–function relationship is claimed?
Imitated property	What function is targeted—lightweighting, energy absorption, friction reduction, thermal control, motion, sensing, or another property?
Digital technology	Which AI, ML, generative AI, LLM, CAD, optimization, CAE, digital twin, IIoT, or robotics function is used?
Material and manufacturing route	Which material and route—AM, molding, machining, forming, surface processing, assembly, or another process—is used or proposed?
Manufacturability	Are repeatability, dimensional tolerance, defect probability, residual stress, fatigue, post-processing time, production rate, yield, or quality control reported?
Scale and production economics	What specimen/batch/field scale is demonstrated, and are CAPEX, OPEX, labor, tooling, cycle time, maintenance, yield, or unit cost reported?
Performance and baseline	What mechanical, thermal, flow, quality, energy, material, or economic metric is reported, against which baseline/control and under which conditions?
Validation and evidence	Is the claim conceptual, digital, laboratory, prototype, pilot/relevant-environment, or serial/commercial, and what replication/uncertainty is reported?
Limitation and Industry 5.0 indicators	Which technical or managerial barrier remains, and are human oversight, safety, life cycle, resilience, adaptability, or cybersecurity outcomes measurable?

**Table 6:** Objective E0–E5 claim-evidence scale and distinction from TRL/MRL.

Level	Minimum Objective Evidence Gate	Exclusion Boundary	Relationship to TRL/MRL (Non-Equivalent)
E0—Conceptual	A stated mechanism, framework, or proposed architecture without demonstrated model or physical performance	No claim of manufacturability or verified performance	May inform early TRL/MRL thinking, but it is a publication-claim category rather than a readiness rating
E1—Digital	A specified CAD/CAE/FEA/CFD, optimization, or digital-twin result with identifiable model assumptions and outcome	No physical specimen or integrated functional test	Can occur at different TRLs/MRLs depending on the system and process; no one-to-one conversion
E2—Laboratory	A physical specimen tested under controlled conditions with a stated comparator or acceptance metric	A one-off or small laboratory test does not establish process repeatability or relevant-environment performance	Evidence strength for a published claim; not a complete system TRL or manufacturing-enterprise MRL
E3—Functional prototype	Integrated components perform the intended function; configuration, test conditions, and functional outcome are reported	No requirement for realistic production volume, sustained field duration, or pilot process capability	Often associated with intermediate readiness, but classification remains case- and claim-specific
E4—Pilot/relevant environment	Integrated pilot in a declared relevant manufacturing/use environment; predefined acceptance criteria; repeated runs or batches; quantified conformance, variation, or reliability; scale and duration reported	Finite pilot evidence only; routine serial production, sustained field continuity, and commercial economics are not yet demonstrated	TRL addresses system technical readiness and MRL manufacturing capability/risk; E4 only records the strength of the reported pilot claim
E5—Serial/commercial	Routine serial production or sustained field/commercial operation across reported batches/deployments and time; documented quality stability or yield/process capability; production economics; qualification, maintenance, or service evidence where applicable	A single pilot, one production lot, short trial, or uncosted field demonstration cannot qualify	Strongest publication-evidence category, but still not a substitute for a formal TRL/MRL assessment by the responsible organization

**Table 7:** (**a**) Biological model, material, manufacturing route, technology, and production scale for the complete Group A subset (*n* = 12). (**b**) Baselines, quantitative performance, validation, evidence level, and major limitations for all Group A studies.

(**a**)						
**Source**	**Biological model/transferred property**	**Material**	**Manufacturing process/route**	**Digital technology**		**Reported production scale**
Gu et al. [21]	Hierarchical natural composites; strength–toughness balance	Printable stiff/soft polymer phases	Multi-material additive manufacture and tensile testing	CNN + FEA		Laboratory tensile coupons; replication number NR
Al Khalil et al. [26]	Plant branching/L-systems; load-adaptive lightweight network	VeroWhite photopolymer	PolyJet material jetting on Objet260 Connex3	Parametric design + FEA		Six configurations designed/printed; no physical mechanical test
Zhang et al. [29]	Tree/branching supports; load-bearing support with less material	Ti-6Al-4V	Selective laser melting (SLM)	Generative design + process simulation		One dental-part case; two proposed supports printed
Efstathiadis et al. [28]	Walnut-shell cells; mechanical interlocking	PLA	Fused-filament fabrication (FFF)	Generative design + FEA		*n* = 3 three-block arrays per reported test orientation
Aruquipa and Diaz [22]	Distributed biological control; local sensing and response	Commercial conveyor/motor and MPU6050 sensor; no biomimetic structural material	Prototype-system integration; component manufacture NR	IoT + autoencoder/LSTM + sensors		One prototype conveyor; 24,000 time points/2400 windows
Kladovasilakis et al. [35]	Earthworm locomotion; flexible multifunctional movement	Dragon Skin 20 silicone; Kevlar reinforcement; VisiJet mold/cover materials	Material-jetting AM mold/core/cover; silicone casting; assembly	FEA + flexible sensor + PID		Integrated actuator/gripper/exoskeleton prototype; cycle test
Kanyilmaz et al. [27]	Natural load paths; material concentration along load paths	SS316L	Metal powder-bed route modeled; supports and heat treatment included	Topology optimization + nonlinear FEA		Digital joint configurations only; no physical build/test
Cadoret et al. [30]	Trabecular bone; directional porosity and lightweighting	PLA	FFF	Parametric model + FEA		Three beam designs; one beam per design appears tested
Wei et al. [34]	Human-skin papillae/mechanoreceptors; conformal multidirectional sensing	CNT/graphene/silicone sensing composite; silver-coated copper/silicone electrodes	Direct extrusion-based multi-material 3D printing	FEA + auxetic/interlocking design		11 × 11 array; six sensors per structure in comparison
Chen et al. [32]	Fish scales; periodic drag-reducing surface	PDMS test surface; aluminum master	Femtosecond laser ablation + PDMS replication	CFD + surface metrology		Five spacings; *n* = 3 per reported test
Díaz Lantada et al. [33]	Tree/fractal branching; stable support with less material	Accura 60 epoxy photopolymer	SLA-3500 photopolymerization	CAD + eco-efficiency analysis		Four printed comparisons; six application geometries total
Arumugam et al. [31]	Honeycomb/spider web/spiral; longer internal heat path	Concrete (modeled)	3D-concrete-printing infill model; no physical print	Rhino 6 + Grasshopper; Ansys Workbench 2022 R2; Ladybug Tools 1.5.0 (Honeybee/Ladybug; EnergyPlus engine)		200 mm simulated cubes and a 3 × 3 × 3 m office model
(**b**)						
**Source**	**Baseline/control**	**Quantitative performance metric**	**Validation method and conditions**	**Statistical treatment**	**E-level**	**Major limitation**
Gu et al. [21]	Stiff constituent, soft constituent, ML-min, and best training design	ML-opt ≈ 25×/40× tougher than stiff/soft phases; ≈2×/>100× stronger; ≈4× tougher than best training design	CNN on ≈100,000 FEA microstructures; printed tensile coupons; toughness from stress–strain area	Error bars shown; n and inferential test NR	E2	Proof-of-concept; quantitative model–experiment agreement and generalizability unverified
Al Khalil et al. [26]	298.2 g rough geometry	74–84% mass reduction; maximum von Mises stress 5.61 MPa compression, 17.04 MPa shear, 32.22 MPa combined	L9 Taguchi DOE; six configurations; 60 N; VeroWhite yield 49.9 MPa; linear FEA	Deterministic DOE/FEA; inferential test NR	E1	No physical load test, repeatability, or process-capability evidence
Zhang et al. [29]	E-stage, Profeta, and Meshmixer supports	1.99–2.06 g versus 3.29, 6.93, and 2.16 g; >40% support saving	Ti-6Al-4V SLM dental part; 45° inclination and 1 mm bridge constraints	Single case; inferential test NR	E2	Surface quality, post-processing, computation, and transfer to other parts unresolved
Efstathiadis et al. [28]	Model 1 versus Model 3	3526.75 ± 193.96 N vs. 1651.85 ± 180.52 N in-plane; 865.50 ± 54.59 N vs. 203.35 ± 10.39 N out-of-plane; <5% FE deviation	*n* = 3; FFF PLA arrays; three-point bending; 5 mm/min	Mean ± SD; inferential test NR	E2	No larger-batch capability, fatigue, tolerance, or field evidence
Aruquipa and Diaz [22]	Standard MQTT and labeled anomaly classes	95.36% accuracy; 89.32% precision; 0.57 s vs. 0.65 s latency (≈12.3% lower; authors state 10%)	Prototype conveyor; 24 V motor; MPU6050; 70/30 split	Single train/test split; CI/inferential test NR	E3	External validation, network scale, maintenance, and cybersecurity untested
Kladovasilakis et al. [35]	Wrapped and embedded reinforcement configurations	14,752 cycles vs. 2846 and 1247; maximum force 7.298 N; manufacture/assembly ≈22 h	Pneumatic actuator; open–close cycling; 500 N load-cell force test; gripper/exoskeleton configurations	Repetitions mentioned; n, dispersion, inferential test NR	E3	Force, yield, miniaturization, repeatability, and relevant-environment validation needed
Kanyilmaz et al. [27]	Unstiffened tubular joints	Linear stress reductions 81.32/76.45% and 82.03/77.68%; at 1700 kN, 72.92/72.52% stress reduction	Four 350 kN load cases; nonlinear load-to-failure; 45° build; supports/heat treatment modeled	Deterministic simulation; inferential test NR	E1	Residual stress, physical strength/fatigue, qualification, and cost unverified
Cadoret et al. [30]	BI2.5D and topology-optimized equal-mass beams	BI3D stiffness 66% and 111% higher at near-equal mass (106.76/103.29/103.29 g)	150 × 30 × 30 mm FFF PLA beams; 130 mm span; least-squares slope	Replication/dispersion and inferential test NR	E2	One beam/design appears; no variation, fatigue, tolerance, or service loading
Wei et al. [34]	Planar sensor	0.63 ± 0.04 vs. <0.03 ± 0.01 kPa^−1^ normal; 0.92 ± 0.08 vs. 0.22 ± 0.05 N^−1^ shear; 0.26 ± 0.03 MPa range; stable 1500 cycles	Six sensors/structure; normal/shear tests; printing on fingertip and bone models	Mean/dispersion; *n* = 6; inferential test NR	E2	Yield, process capability, drift, and relevant-environment durability unreported
Chen et al. [32]	Smooth PDMS and wider scale spacings	Maximum 10.26% drag reduction at Re = 39,532	Five spacings 42–994 μm; water tunnel 0.5–2 m/s at 25 ± 2 °C; *n* = 3	Averages/error bars; inferential test NR	E2	Durability, fouling, surface tolerance, manufacturing rate, and scale unresolved
Díaz Lantada et al. [33]	Formlabs and 3D Lightyear supports	Material saving 55–80% and 12–47%; cost saving 22–42% and 1–16%	Four printed geometries; measured mass/time; energy/cost equations	5% uncertainty bars; inferential test NR	E2	Process/material-specific; not full LCA or volume-sensitive total-cost model
Arumugam et al. [31]	Solid concrete and sawtooth patterns	Spiral U-value 1.22 vs. 2.30 W m^−2^ K^−1^ (≈47% lower); annual energy <600 vs. 965 kWh (≈43% lower); 25–40% modeled material saving	ANSYS at 40/22 °C; EnergyPlus Kuala Lumpur office scenario	Scenario modeling; inferential test NR	E1	No physical printing, structural safety, climate transfer, or whole-life validation

**Table 8:** Production-route-neutral scaling bottlenecks and evidence required for industrial transition.

Bottleneck	Pattern in the Literature	Evidence Required for Industrial Transition
Biological abstraction	The source model is often described visually	Structure–function causality, traceable requirements, and controlled comparator
Data/AI	Fragmented datasets; limited explainability, uncertainty, and model updating	Cross-scale data, external validation, uncertainty analysis, human oversight, and data governance
Computation/digital validation	Optimization and high-fidelity simulation can be slow or detached from process data	Calibrated surrogates, model–experiment agreement, computing budget, and update rules
Manufacturing process capability	Repeatability, tolerance, defect probability, residual stress, fatigue, post-processing time, production rate, yield, and quality control are rarely reported together	Qualified process window, measurement-system analysis, DOE/SPC, capability indices or conformance rates, fatigue and defect maps, and route-specific QC
Quality/standards	Laboratory performance dominates; acceptance and qualification are incomplete	Acceptance criteria, traceability, certification, multi-batch reliability, and field testing
Production economics	CAPEX, OPEX, cycle time, labor, yield loss, tooling, post-processing, maintenance, and unit cost are seldom reported across volume scenarios	Transparent total-cost model, volume sensitivity, learning/yield assumptions, capacity and maintenance data
Sustainability	Material reduction is common; functional-unit LCA and service-life evidence are rare	Comparative energy, waste, carbon, repair, lifetime, and end-of-life data
Operations/deployment	Sensors and feedback remain prototypical; interoperability and cyber risk are under-tested	IIoT integration, field availability, recovery/reconfiguration, maintenance, cybersecurity, and multi-site testing

Synthesis based on Lu et al. [5], Ambaye et al. [42], Mirhakimi et al. [43], Favi et al. [63], and Petrillo et al. [38].

**Table 9:** Consolidated quantitative findings from direct Group A studies.

Outcome Domain	Direct Studies (E-Level)	Consolidated Quantitative Finding	Validation Basis and Inferential Boundary
Mechanical strength, toughness, and stiffness	[21] E2; [28] E2; [27] E1; [30] E2	ML-designed composites reached ≈25–40× constituent toughness and ≈2× stiff-phase strength [21]; interlocking blocks carried 3526.75 ± 193.96 N in-plane [28]; simulated joint stresses fell ≈76–82% [27]; a trabecular-bone beam was 66% and 111% stiffer than equal-mass comparators [30].	Three physical laboratory studies and one simulation study; heterogeneous units, baselines, replication, and missing inferential tests preclude pooling.
Mass, support material, and production cost	[26] E1; [29] E2; [33] E2	Reported mass reductions were 74–84% in digital lightweight designs [26]; SLM support mass was 1.99–2.06 g versus 3.29–6.93 g for two comparators [29]; SLA supports saved 55–80%/12–47% material and 22–42%/1–16% cost versus two software baselines [33].	One digital and two physical case sets; cost boundaries are process-specific and not volume-sensitive enterprise economics.
Flow, thermal, and energy performance	[32] E2; [31] E1	A fish-scale surface produced 10.26% maximum drag reduction at Re = 39,532 [32]. A modeled spiral concrete infill reduced the U-value from 2.30 to 1.22 W m^−2^ K^−1^ and the annual energy from 965 to <600 kWh [31].	Controlled water-tunnel test versus simulation-only building scenarios; no cross-domain average effect is meaningful.
Sensing, monitoring, and response	[22] E3; [34] E2	The conveyor prototype reported 95.36% anomaly classification accuracy and 0.57 s latency [22]. The tactile sensor achieved 0.63 ± 0.04 kPa^−1^ normal and 0.92 ± 0.08 N^−1^ shear sensitivity, with a stable response over 1500 cycles [34].	One integrated prototype and one laboratory sensor study; external validation, yield, drift, and network-scale performance remain open.
Actuation durability and production time	[35] E3	A Kevlar–silicone configuration endured 14,752 cycles versus 2846 and 1247 for two alternatives; maximum force was 7.298 N and reported manufacturing/assembly time was ≈22 h.	Integrated functional prototype; sample count, dispersion, process repeatability, unit cost, and relevant-environment life were not reported.

The largest reported gains concern component-level outcomes measured under study-specific conditions. They support technical feasibility and comparative performance, but they do not establish average effects across a population of studies, repeatability over multiple batches, or the economics of serial production.

**Table 10:** Measurable Industry 5.0 indicators for biomimetic industrialization.

Industry 5.0 Criterion	Measurable Indicators	Example Unit or Decision Rule	Interpretive Boundary
Human oversight and accountability	Defined decision authority, review/override rate, model explanation coverage, audit-trail completeness	% AI recommendations reviewed/overridden; named approval role; traceable change record	AI assistance alone is not human-centric without documented authority and accountability
Worker safety and ergonomics	Incident/near-miss rate, hazardous exposure, ergonomic load, safe-stop performance, human–robot separation	Incidents per hour worked; exposure dose; RULA/REBA; stop time/distance	A soft or bio-inspired robot is not inherently safe
Energy performance	Build/use energy and peak demand per functional output	kWh per conforming part, service hour, or functional unit	Lower mass does not establish lower total energy
Material efficiency	Material yield, scrap/support/rework fraction, recycled content, conformance yield	kg input/kg conforming output; first-pass yield; % scrap/support	Material saving must include failed builds and post-processing losses
Life-cycle impact	GWP and other impacts per functional unit; service life; repairability; end-of-life recovery	kg CO_2_-eq per functional unit; years/cycles; repair time; recovery rate	Biological derivation is not evidence of net sustainability
Resilience and adaptability	Availability, recovery time, reconfiguration time, performance retained under disturbance	% availability; MTTR; time to reconfigure; % function retained	Modularity or adaptability is only potential until disruption scenarios are tested
Cybersecurity and data integrity	Authentication coverage, data/model integrity checks, anomaly detection time, fail-safe and recovery performance	% authenticated assets; detection/recovery time; tested fail-safe state	Connected sensing/digital twins add cyber–physical risk that must be included in deployment evidence

**Table 11:** Decision gates and supporting studies in the transformation framework.

Stage	Principal Question	Transition Evidence	Supporting Studies in the Selected Set
1. Biological model	What function is imitated, and through which mechanism?	Source and structure–function evidence	[4,5,6,7,8,9,10,11,12,18,19,21,22,23,24,25,26,27,28,29,30,31,32,33,34,35,39,40,41,42,43,53,54,55,56,57,58,59,60,61,62,64]
2. Abstraction	Which technical variables represent the function?	Requirements and traceability	[4,5,6,7,8,9,10,11,12,18,19,21,22,23,24,25,26,27,28,29,30,31,32,33,34,35,39,40,41,42,43,53,54,55,56,57,58,59,60,61,62]
3. Digital design	How is the solution generated under material and manufacturing constraints?	Parametric model, constraints, and design rules	[4,5,9,10,11,12,18,19,20,21,23,24,25,26,27,28,29,30,31,32,33,34,35,39,40,41,42,43,53,54,55,56,57,58,59,60,61,62,63,64]
4. Virtual validation	How confidently does the model represent real behavior and process variation?	Model–experiment agreement, sensitivity, and uncertainty	[4,5,9,11,12,18,19,20,21,23,24,25,26,27,28,29,30,31,32,33,34,35,40,42,43,53,54,55,56,57,58,59,60,61,62]
5. Physical manufacturing	Which production route can reproduce the critical function within a controlled process window?	Process plan, tolerance/defect control, repeatability, rate, and specimen validation	[4,5,6,8,9,10,18,20,21,26,27,28,29,30,31,32,33,34,35,37,40,41,42,43,55,56,57,58,59,60,61,62,63]
6. Functional validation	Is performance maintained against explicit acceptance criteria?	Tests, sensor data, reliability, and acceptance evidence	[5,9,10,11,12,20,21,22,25,27,28,29,30,31,32,33,34,35,36,37,38,39,40,41,42,43,45,46,47,53,54,55,56,57,58,59,60,61,62,64]
7. Industrial scaling	Are function, quality, rate, economics, and environmental value preserved across volume, site, and time?	Pilot/serial evidence, qualification, total cost, LCA, field and maintenance data	[4,5,6,7,8,9,10,11,12,18,20,22,27,31,33,36,37,38,39,40,41,42,43,45,46,47,53,56,63,64]

**Table 12:** Author-derived theoretical propositions and their supporting evidence.

Proposition	Mechanism	Expected Outcome	Supporting Evidence in the Selected Set
P1—Transfer integrity	The biological structure–function relationship is translated into traceable technical requirements	Less visual imitation and more verifiable function	Direct: [21] E2; [26] E1; [28] E2; [27] E1; [29] E2; [30] E2; [32] E2; [33] E2; [31] E1; [35] E3; [34] E2. Context: [4,5,6,7,8,9,41,54,55,56,57,58,59,60,61].
P2—Manufacturing-constrained digital design	AI/generative design incorporates material and process constraints early	Less redesign and fewer manufacturing failures	Direct: [21] E2; [26] E1; [28] E2; [27] E1; [29] E2; [30] E2; [33] E2; [31] E1. Context: [4,5,9,10,18,19,20,23,24,43,54,55,56,57,58,59,60,61,63].
P3—Evidence alignment	Digital, experimental, and process evidence share one functional acceptance criterion	Easier transition from laboratory to pilot scale	Direct: [21] E2; [28] E2; [29] E2; [30] E2; [32] E2; [33] E2; [35] E3. Context: [5,9,10,20,40,42,43,56,61].
P4—Closed-loop learning	Sensor/IIoT and digital-twin data update the design model	Stronger quality stability and adaptability	Direct: [22] E3; [35] E3; [34] E2. Context: [12,36,37,38,62,64].
P5—Industry 5.0 governance	Human oversight, LCA, and resilience criteria guide technology decisions	Technical performance becomes more responsible industrial value	Direct: [33] E2; [31] E1; [35] E3. Context: [6,7,8,11,18,36,37,38,39,45,46,47,63,64].

**Table 13:** Future research agenda.

Research Area	Priority Question	Recommended Evidence
Biological knowledge and AI	How can biological mechanisms be matched to manufacturing constraints while preserving human accountability?	Open datasets, ontologies, external validation, XAI, uncertainty, and expert review
Digital twins	How can biological principles, process, quality, and field-performance data form a closed loop?	Real-time pilots, model updating, data integrity and cybersecurity tests
Manufacturability	How do route-specific variations affect biomimetic function?	DOE/SPC, tolerance and defect maps, residual stress, fatigue, post-processing time, production rate, yield, and quality-control capability
Production economics	At what volume and capacity utilization is each manufacturing route advantageous?	CAPEX/OPEX, tooling, labor, cycle time, yield/rework, post-processing, maintenance, unit cost, and volume sensitivity
Sustainability	Does material reduction create net environmental value over the functional life?	Comparative LCA, energy/material balance, service life, repair, and end-of-life data
Human-centricity and safety	How do AI and robotics affect authority, workload, competence, and risk?	Field studies; audit/override measures; ergonomic, exposure, incident, trust, and decision-right indicators
Resilience and adaptability	Does bio-inspired modularity improve performance under disruption?	Failure scenarios, availability, recovery and reconfiguration time, and cyber–physical resilience tests

## Data Availability

The bounded search audit, bibliographic trail of the 50 selected studies, expanded coding dictionary, objective evidence-level definitions, complete Group A evidence table, technology–evidence matrix, quantitative synthesis, study-stage matrix, and proposition–evidence map are provided in Supplementary File S1. Article PDFs and proprietary Web of Science export files are not redistributed.

## Supplementary Materials

The following supporting information can be downloaded at https://www.mdpi.com/article/10.3390/biomimetics11090614/s1, Supplementary File S1, “Search Audit, Bibliographic Trail, Coding Dictionary, Evidence Tables, and Evidence Maps,” contains the search-audit flow and limitations, the 50-study bibliographic trail, expanded coding dictionary, objective E0–E5 definitions, complete Group A evidence table, technology–E-level matrix, consolidated quantitative synthesis, 50-study × seven-stage framework matrix, and proposition–evidence map. Article PDFs and proprietary database exports are not redistributed.

## Appendix A. Summary of the Web of Science Search and Evidence Set

The Web of Science Core Collection was searched on 3 August 2026 across the fourteen concept families listed in Section 3.2 and Supplementary File S1. Gold Open Access, Article/Review Article, and English filters formed operational boundaries. WoS Excel exports were manually screened within each query family using relevance ranking, title/abstract/keyword fit, citation influence, and recency; highly cited foundational and recent 2023–2026 records were assessed before full-text eligibility and maximum-variation selection. Exact Smart Search strings and a record-level exclusion log were not retained, so stepwise exclusion counts are not reconstructed. The auditable downstream flow is 52 acquired PDFs, removal of two duplicate copies, and final *n* = 50 (A = 12, B = 25, C = 13). Supplementary File S1 records the criteria, audit boundary, and study-level evidence maps.

## References

[1] Vincent J.F.V., Bogatyreva O.A., Bogatyrev N.R., Bowyer A., Pahl A.-K. (2006). Biomimetics: Its practice and theory. J. R. Soc. Interface.

[2] Helms M., Vattam S.S., Goel A.K. (2009). Biologically inspired design: Process and products. Des. Stud..

[3] Shu L.H., Ueda K., Chiu I., Cheong H. (2011). Biologically inspired design. CIRP Ann.-Manuf. Technol..

[4] Gralow M., Weigand F., Herzog D., Wischeropp T., Emmelmann C. (2020). Biomimetic design and laser additive manufacturing—A perfect symbiosis?. J. Laser Appl..

[5] Lu J.-H., Ding S., Ni Y.-Q., Li S. (2025). Bio-inspired acoustic metamaterials for traffic noise control: Bridging the gap with machine learning. Commun. Eng..

[6] Byrne G., Dimitrov D., Monostori L., Teti R., van Houten F., Wertheim R. (2018). Biologicalisation: Biological transformation in manufacturing. CIRP J. Manuf. Sci. Technol..

[7] Thielen M., Trube N., Schneider J.M., von Ramin M. (2024). Biomimetic Regulation in Supply Chains and Production Systems. Adv. Intell. Syst..

[8] Unruh G.C. (2023). From Geomimetic to Biomimetic Manufacturing: Digitally Transforming Industry for Sustainability. Sustainability.

[9] du Plessis A., Babafemi A.J., Paul S.C., Panda B., Tran J.P., Broeckhoven C. (2021). Biomimicry for 3D concrete printing: A review and perspective. Addit. Manuf..

[10] Yan X.X., Bethers B., Chen H.X., Xiao S.Q., Lin S., Tran B., Jiang L.M., Yang Y. (2021). Recent Advancements in Biomimetic 3D Printing Materials with Enhanced Mechanical Properties. Front. Mater..

[11] Gai M., Kam K.J., Flor J.-F., Chai C., Gunasagaran S. (2026). A Generative AI Framework for Carbon-Oriented Biomimetic Façade Design in Architecture. Buildings.

[12] Li L.L., Gu F., Li H., Guo J.F., Gu X.J. (2021). Digital Twin Bionics: A Biological Evolution-Based Digital Twin Approach for Rapid Product Development. IEEE Access.

[13] Shen T., Li N., Liu S., Yu C., Zhang C., Yang K., Li X., Fang R., Jiang L., Dong Z. (2024). Fast prototype and rapid construction of three-dimensional and multi-scaled pitcher for controlled drainage by systematic biomimicry. Int. J. Extrem. Manuf..

[14] Shu Y., Chen J., Xu B., Liu Z., Zheng H., Zhang F., Fu W. (2024). Biomimetic Synthesis of Nanosilica by Deep Learning-Designed Peptides and Its Anti-UV Application. Adv. Intell. Syst..

[15] Speck O., Speck D., Horn R., Gantner J., Sedlbauer K.P. (2017). Biomimetic bio-inspired biomorph sustainable? An attempt to classify and clarify biology-derived technical developments. Bioinspir. Biomim..

[16] (2015). Biomimetics—Terminology, Concepts and Methodology.

[17] (2016). Biomimetics—Biomimetic Materials, Structures and Components.

[18] Stefanska A., Kurcjusz M. (2025). From Nature to Neutral Networks: AI-Driven Biomimetic Optimization in Architectural Design and Fabrication. Sustainability.

[19] Marom L., Buehler M.J. (2025). Frontiers of biological material intelligence. MRS Bull..

[20] Zaman S., Mahmud M.S., Mollick A.A., Lhaden T., Dantzler J., Arroyo S., Goona N.K., Mesbah M., Ahsan M.A., Lin Y.R. (2026). Artificial intelligence in additive manufacturing: Advances in smart materials, lattice optimization, and process intelligence. Int. J. Adv. Manuf. Technol..

[21] Gu G.X., Chen C.T., Richmond D.J., Buehler M.J. (2018). Bioinspired hierarchical composite design using machine learning: Simulation, additive manufacturing, and experiment. Mater. Horiz..

[22] Aruquipa G., Diaz F. (2022). An IoT architecture based on the control of Bio Inspired manufacturing system for the detection of anomalies with vibration sensors. Procedia Comput. Sci..

[23] Deng Z.G., Lv J., Liu X., Hou Y.K. (2023). Bionic Design Model for Co-creative Product Innovation Based on Deep Generative and BID. Int. J. Comput. Intell. Syst..

[24] Badini S., Regondi S., Pugliese R. (2025). Enhancing mechanical and bioinspired materials through generative AI approaches. Next Mater..

[25] Luu R.K., Buehler M.J. (2024). BioinspiredLLM: Conversational Large Language Model for the Mechanics of Biological and Bio-Inspired Materials. Adv. Sci..

[26] Al Khalil M., Belkebir H., Lebaal N., Demoly F., Roth S. (2022). A Biomimetic Design Method for 3D-Printed Lightweight Structures Using L-Systems and Parametric Optimization. Appl. Sci..

[27] Kanyilmaz A., Berto F., Paoletti I., Caringal R.J., Mora S. (2021). Nature-inspired optimization of tubular joints for metal 3D printing. Struct. Multidiscip. Optim..

[28] Efstathiadis A., Symeonidou I., Tsongas K., Tzimtzimis E.K., Tzetzis D. (2026). Generative Design of 3D-Printed Biomimetic Interlocking Blocks Inspired by the Cellular 3D Puzzle Structure of the Walnut Shell. Biomimetics.

[29] Zhang Y.C., Wang Z.P., Zhang Y.C., Gomes S., Bernard A. (2020). Bio-inspired generative design for support structure generation and optimization in Additive Manufacturing (AM). CIRP Ann..

[30] Cadoret N., Chaves-Jacob J., Linares J.M. (2023). Structural additive manufacturing parts bio-inspired from trabecular bone form-function relationship. Mater. Des..

[31] Arumugam G., Kusumo C.M.L., Mari T.S. (2025). Impact of Bioinspired Infill Pattern on the Thermal and Energy Efficiency of 3D Concrete Printed Building Envelope. Architecture.

[32] Chen D.K., Zhang B.W., Zhang H.F., Shangguan Z., Sun C.G., Cui X.X., Liu X.L., Zhao Z.H., Liu G., Chen H.W. (2024). Laser Ablating Biomimetic Periodic Array Fish Scale Surface for Drag Reduction. Biomimetics.

[33] Díaz Lantada A., Romero A.D., Isasi A.S., Bellido D.G. (2017). Design and Performance Assessment of Innovative Eco-Efficient Support Structures for Additive Manufacturing by Photopolymerization. J. Ind. Ecol..

[34] Wei Y., Li B., Domingos M., Qian Z., Zhu Y., Yan L., Ren L., Wei G. (2023). Fully 3D printed flexible, conformal and multi-directional tactile sensor with integrated biomimetic and auxetic structure. Commun. Eng..

[35] Kladovasilakis N., Sideridis P., Tzetzis D., Piliounis K., Kostavelis I., Tzovaras D. (2022). Design and Development of a Multi-Functional Bioinspired Soft Robotic Actuator via Additive Manufacturing. Biomimetics.

[36] Siafara L.C., Kholerdi H., Bratukhin A., Taherinejad N., Jantsch A. (2018). SAMBA—An architecture for adaptive cognitive control of distributed Cyber-Physical Production Systems based on its self-awareness. E I Elektrotech. Inf..

[37] Richard D., Jang J., Itmaci B.C., Luo J.W., Canuso V., Korambath P., Morales-Leslie O., Davis J.F., Malkani H., Christofides P.D. (2023). Smart manufacturing inspired approach to research, development, and scale-up of electrified chemical manufacturing systems. iScience.

[38] Petrillo A., Caiazzo B., Piccirillo G., Santini S., Murino T. (2023). An IoT-based and cloud-assisted AI-driven monitoring platform for smart manufacturing: Design architecture and experimental validation. J. Manuf. Technol. Manag..

[39] Urrea C. (2025). Artificial Intelligence-Driven and Bio-Inspired Control Strategies for Industrial Robotics: A Systematic Review of Trends, Challenges, and Sustainable Innovations Toward Industry 5.0. Machines.

[40] Bliah O., Hegde C., Tan J.M.R., Magdassi S. (2025). Fabrication of Soft Robotics by Additive Manufacturing: From Materials to Applications. Chem. Rev..

[41] Speck T., Cheng T., Klimm F., Menges A., Poppinga S., Speck O., Tahouni Y., Tauber F., Thielen M. (2023). Plants as inspiration for material-based sensing and actuation in soft robots and machines. MRS Bull..

[42] Ambaye G., Boldsaikhan E., Krishnan K. (2024). Soft Robot Design, Manufacturing, and Operation Challenges: A Review. J. Manuf. Mater. Process..

[43] Mirhakimi A.S., Dubey D., Elbestawi M.A. (2024). Laser powder bed fusion of bio-inspired metamaterials for energy absorption applications: A review. J. Mater. Res. Technol..

[44] Breque M., De Nul L., Petridis A. (2021). Industry 5.0: Towards a Sustainable, Human-Centric and Resilient European Industry.

[45] Turner C., Oyekan J. (2023). Manufacturing in the Age of Human-Centric and Sustainable Industry 5.0: Application to Holonic, Flexible, Reconfigurable and Smart Manufacturing Systems. Sustainability.

[46] Djebbouri K., Alofaysan H., Hassan F.A., Mohammed K.S. (2025). Industry 5 Digital DNA: A Genetic Code of Human-Centric Smart Manufacturing. Sustainability.

[47] Wang B.C., Tao F., Fang X.D., Liu C., Liu Y.F., Freiheit T. (2021). Smart Manufacturing and Intelligent Manufacturing: A Comparative Review. Engineering.

[48] Snyder H. (2019). Literature review as a research methodology: An overview and guidelines. J. Bus. Res..

[49] Jaakkola E. (2020). Designing conceptual articles: Four approaches. AMS Rev..

[50] Zeng S., Zhang R., Cai Y. (2024). Research on Digital Morphogenesis and Sustainability of 3D Printing Bionic Materials Based on Convolutional Neural Networks. IEEE Access.

[51] National Aeronautics and Space Administration Technology Readiness Levels. https://www.nasa.gov/directorates/somd/space-communications-navigation-program/technology-readiness-levels/.

[52] Office of the Secretary of Defense Manufacturing Technology Program, Joint Service/Industry Manufacturing Readiness Level Working Group (2025). Manufacturing Readiness Level (MRL) Deskbook, Version 2025.

[53] Yang F., Shuai C., Feng P. (2026). Biomimetic additive manufacturing of gradient cellular solids inspired by abiotic mechanoelectrical perception. Int. J. Extrem. Manuf..

[54] Ren Y., Lv B., Ran Z. (2026). Application of Computational Fluid Dynamics—Optimized Biomimetic Microchannel Liquid-Cooled Plates in Battery Thermal Management Systems. J. Appl. Fluid Mech..

[55] Efstathiadis A., Symeonidou I., Tsongas K., Tzimtzimis E.K., Tzetzis D. (2023). Parametric Design and Mechanical Characterization of 3D-Printed PLA Composite Biomimetic Voronoi Lattices Inspired by the Stereom of Sea Urchins. J. Compos. Sci..

[56] Ryan-Johnson W.P., Wolfe L.C., Byron C.R., Nagel J.K., Zhang H. (2021). A Systems Approach of Topology Optimization for Bioinspired Material Structures Design Using Additive Manufacturing. Sustainability.

[57] Yin L., Jiaqiang E., Tu Y., Luo W.H. (2025). Biomimetic microchannel structures and their topological optimization: A review. Int. Commun. Heat Mass Transf..

[58] Islam M.K., Hazell P.J., Escobedo J.P., Wang H.X. (2021). Biomimetic armour design strategies for additive manufacturing: A review. Mater. Des..

[59] Tee Y.L., Maconachie T., Pille P., Leary M., Do T., Tran P. (2021). From nature to additive manufacturing: Biomimicry of porcupine quill. Mater. Des..

[60] Podrouzek J., Marcon M., Nincevic K., Wan-Wendner R. (2019). Bio-Inspired 3D Infill Patterns for Additive Manufacturing and Structural Applications. Materials.

[61] Siddique S.H., Hazell P.J., Wang H.X., Escobedo J.P., Ameri A.A.H. (2022). Lessons from nature: 3D printed bio-inspired porous structures for impact energy absorption-A review. Addit. Manuf..

[62] Kubota K., Tanaka H. (2025). Machine Learning-Based Wind Classification by Wing Deformation in Biomimetic Flapping Robots: Biomimetic Flexible Structures Improve Wind Sensing. Adv. Intell. Syst..

[63] Favi C., Murgese L., Gallozzi S., Chiacchietta C., Marconi M., Mandolini M. (2025). Definition of a life cycle engineering tool to support the ecodesign in additive manufacturing: Application to FDM technology. Int. J. Interact. Des. Manuf..

[64] Eraghi S.H., Toofani A., Aghamoali A., Zoroufi A., Shafaghi M., Jafari S., Goel S., Rajabi H. (2026). The rise of metaverse manufacturing: Towards a universal, sustainable and bioinspired industrial ecosystem. J. R. Soc. Interface.

